# Metagenome Sequencing Reveals the Microbiome of *Aedes albopictus* and Its Possible Relationship With Dengue Virus Susceptibility

**DOI:** 10.3389/fmicb.2022.891151

**Published:** 2022-05-11

**Authors:** Teng Zhao, Bo-qi Li, He-ting Gao, Dan Xing, Man-jin Li, Yun-qi Dang, Heng-duan Zhang, Yue-e Zhao, Zhu Liu, Chun-xiao Li

**Affiliations:** ^1^State Key Laboratory of Pathogens and Biosecurity, Institute of Microbiology and Epidemiology, Beijing, China; ^2^College of Life Science and Technology, Mudanjiang Normal University, Mudanjiang, China

**Keywords:** *Aedes albopictus*, microbiome, dengue virus, metagenome, susceptibility

## Abstract

Dengue fever virus (DENV) is a mosquito-borne flavivirus that poses a serious risk to human health. *Aedes albopictus* is a widely distributed vector of dengue fever in China. Based on the impact of physiological activity, the microbiome in *A. albopictus* will provide a novel environment-friendly approach to control DENV transmission. We performed metagenomic sequencing on *A. albopictus* before and after exposure to DENV blood meal to detect microbiome variation of *A. albopictus* with different susceptibilities to DENV. The dominant phyla in *A. albopictus* microbiome were *Proteobacteria* and *Ascomycota*, and the dominant genera were *Aspergillus* and *Metarhizium*. *Gammaproteobacteria bacterium*, *Lactobacillus harbinensis*, and *Neurospora crassa* differed significantly after DENV infection. There were 15 different microorganisms found to be involved in mosquito immunity and metabolism, such as *Alphaproteobacteria bacterium*, *Methyloglobulus morosus*, and *Shigella sonnei*, which might have an impact on the DENV susceptibility of *A. albopictus*. It was hypothesized that the lack of specific bacteria may lead to increased susceptibility of *A. albopictus* to DENV. Interventions in the microbiome composition or specific bacteria of *A. albopictus* may affect the susceptibility to DENV and control the mosquito-borne diseases efficiently.

## Introduction

Dengue fever virus (DENV) is a mosquito-borne flavivirus that poses a serious threat to human health. In recent years, the global incidence of dengue fever has been increasing, with approximately 390 million infections per year, including nearly 20,000 deaths (Guzman et al., 2010; Guo et al., 2017). In 2013, the local outbreaks of dengue fever occurred in Guangdong and Yunnan provinces of China, resulting in thousands of infections (Luo et al., 2017; Sun et al., 2020).

*Aedes albopictus*, known as the “Asian tiger mosquito,” is the most dominant species and is found in nearly one-third of China. Both *A. albopictus* and *Aedes aegypti* are two important vector species for dengue transmission in China (Souza-Neto et al., 2019), but *A. albopictus* is more widely distributed. *A. albopictus* is reported to be the only mosquito vector for dengue transmission in Guangdong, and *A. aegypti* has not been detected there in the last 30 years. The strong adaptability of *A. albopictus* contributed to the re-emergence and widespread spread of dengue fever in China (Ruiling et al., 2018; Wei et al., 2021).

Dengue viruses belong to the genus *Flavivirus* in the family Flaviviridae and are classified into four serotypes (Gebhard et al., 2011; Vasilakis et al., 2011), with DENV2 being the most transmissible and virulent (Guzman and Kouri, 2003; Balmaseda et al., 2006). Humans, who once infected with a specific serotype of DENV, can only produce the corresponding antibodies which cannot protect against all four serotypes simultaneously (Deng et al., 2020). Due to the lack of an effective vaccine or drug, mosquito control remains the primary means of prevention and control of DENV. There is an urgent need to find a new environmentally friendly mosquito control technology (Dimopoulos, 2019; Zheng et al., 2019).

The mosquito microbiome is extremely large and diverse, with important roles in infection, immunity, nutrition, and physiological behavior (Minard et al., 2013). Increasingly, this research is focusing on the impact of microbes on vector competence to control mosquito-borne diseases. For example, *Asaia* was a stable and dominant bacterium in mosquitoes that can compete with *Wolbachia* to negatively affect the growth and development, thereby reducing mosquito populations (Rossi et al., 2015). *Leptolegnia chapmanii Seymour* exhibited the significant pathogenicity to larvae (Pelizza et al., 2013). *Serratia marcescens* was confirmed to digest the membranous mucin of the intestine, thus facilitating virus transmission (Wu et al., 2019). In addition, mosquito symbiotic microbes ultimately influence the efficiency of pathogen transmission by regulating the full cycle of the larval development rate, pupation time, adult fecundity, and survival rate (Yee et al., 2012; Souza et al., 2019; Buxton et al., 2020).

We designed the experiment using metagenome sequencing approaches to compare the microbiomes of *A. albopictus* with different susceptibility to DENV2. By analyzing the diversity of their microbiome composition, differences in abundance, annotated gene functions, and other biological information, we also established the association between the symbiotic microbiome of *A. albopictus* and DENV2 susceptibility. Artificially interfering with the mosquito microbiome may indirectly influence the susceptibility of mosquitoes, providing a new technical means to control the DENV2 transmission by *A. albopictus*.

## Materials and Methods

### Mosquito

The *A. albopictus* strain was originally collected in 2019 in Guangzhou, Guangdong Province (112°57′ to 114°3′E, 22°26′ to 23°56′N). Mosquitoes were reared at 26 ± 1°C, 75 ± 5% relative humidity and a 14:10 h light cycle, and larvae were provided with rat food. Cultures were maintained by feeding adult mosquitoes with 8% sugar water. Adult female mosquitoes used for DENV infection were 3–5 days post-emergence.

### Viral and Normal Blood Meals for Mosquitoes

Dengue serotype 2 was inoculated into the brains of SPF (specific–pathogen-free) mice and cultured. Approximately 5 days after inoculation, mice were dissected to obtain DENV-infected brain suspensions. The supernatant was centrifuged at 8,000 rpm for 10 min and then filtered through 0.45 and 0.2 μm membranes. DENV2 suspension was diluted with Dulbecco’s modified eagle medium (DMEM) to 1 × 10^6^ plaque forming units (pfu)/ml and then mixed with SPF mice blood at a 1:1 concentration as DENV2 viral blood meal. The normal blood meal for the control group was DMEM to blood at a ratio of 1:1. The blood meal cups were soaked in 1% sodium heparin to avoid clotting. Blood meal cups providing the viral and normal blood meals were then sealed with film and attached to a thermostatic blood donor simulator. They were placed in cages containing approximately 500 mosquitoes that were starved 18 h in advance for 2 h. By observing the degree of abdominal expansion, virus blood-fed and normal blood-fed female mosquitoes were picked out and transferred to other cages separately. All mosquitoes were reared at 26 ± 1°C, 75 ± 5% relative humidity and a 14:10 h light cycle.

### Detection and Grouping of Mosquito Infections

Approximately 7 days after blood feeding, DENV2 infection was detected in viral blood-fed and normal blood-fed female mosquitoes using an RNA extraction kit (QIAGEN Shanghai, China) and a Mylab dengue virus type II nucleic acid detection kit (Mylab Inc., Beijing, China). The PCR procedure was as follows: 50°C for 30 min, 95°C for 15 min, followed by 40 cycles of 95°C for 15 s and 60°C for 45 s. Finally, the mosquitoes were divided into three groups. Mosquitoes that fed on the viral blood meal and tested positive were defined as the infected group. Mosquitoes that fed on the viral blood meal and tested negative were defined as the uninfected group. Mosquitoes that consumed a normal blood meal were defined as the control group. Mosquitoes were randomly selected and replicated three times with 20 mosquitoes for each group.

### Library Construction and Sequencing

Mosquitoes were washed three times with 75% ethanol and 1× phosphate buffer to avoid environmental bacterial contamination. Microbial genomic DNA was extracted using QIAamp DNA Microbiome Kit (Shanghai, China). Samples were tested for concentration, total amount, purity and degradation, and then sent to Biomark Technologies (Beijing, China) for metagenomic sequencing. After the genomic DNA of the samples passed quality testing, the DNA was fragmented by the mechanical shearing (ultrasonic treatment). The fragmented DNA is then purified, end-repaired, 3′-adenylated, ligated to sequencing adapters, and then electrophoresed in agarose gels for fragment size selection. The Illumina sequencing platform was used to perform the metagenomic sequencing on qualified libraries.

### Bioinformatics Analysis

The raw data were filtered by the Trimmomatic software (Bolger et al., 2014), and the clean reads were compared to the reference genome to remove host using bowtie2 software (Langmead and Salzberg, 2012), reference genome file acquired from the National Center for Biotechnology Information (NCBI)^1^ (Chen et al., 2015; O’Leary et al., 2016). Metagenome assembly were conducted with megahit software (Li et al., 2016) and quality assessment of assembled contigs were conducted with QUAST software (Gurevich et al., 2013). Species annotated were using kraken2 software (Wood et al., 2019), while the bracken software was used to estimate the relative abundances within a specific sample from Kraken 2 classification results (Lu et al., 2017). Functional annotations are annotated using databases such as Kyoto Encyclopedia of Genes and Genomes (KEGG) (Kanehisa et al., 2022), non-redundant protein (Nr), Gene Ontology (GO) (Mi et al., 2019, Gene Ontology Consortium, 2021), etc. Differential species analysis was performed using the R package metagenomeSeq. The screening criteria for differentially expressed species were padj < 0.05 and | log2 (fold change) | ≥ 1. The random forest analysis uses the R package random Forest to calculate the optimal value of the model capacity through 10-fold cross-validation (Liaw and Wiener, 2001).

## Results

### Sequencing Data Quality

After sample data were filtered and the host sequences were removed, the metagenomic sequencing depth of 9 samples was around 30 G. The number of valid reads ranged from 37,348,805 to 47,179,819. The Q20 values were all above 95% and N50 values were above 700 bp, suggesting a high degree of gene assembly integrity and quality (Supplementary Table 1). The species cumulative curve showed that the curve rose sharply as the sample size increased, indicating that a large number of species were found. The curve smoothed indicating the number of species reached saturation and the sample size was sufficient to contain most of the species at this time (Supplementary Figure 1).

### Microbial Community Composition in *Aedes albopictus*

**FIGURE 1:**
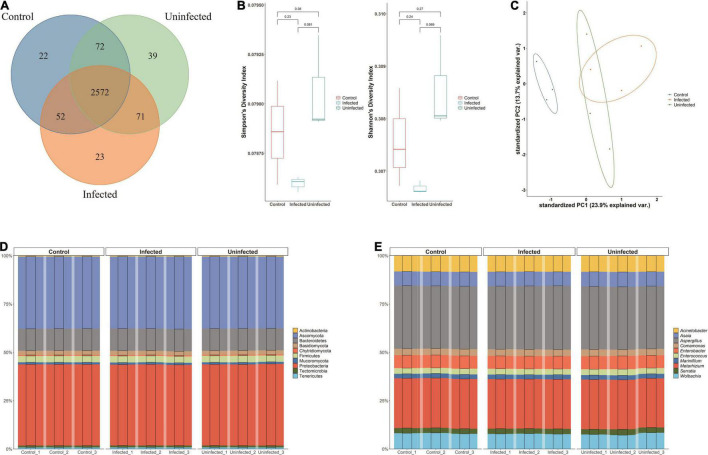
Microbiota of *Aedes albopictus*. **(A)** Venn diagram of the overlapped species among DENV2-infected, DENV2-uninfected, and control group. **(B)** Within-sample diversity (alpha-diversity) with Shannon and Simpson index among three groups. The horizontal bars within boxes represent medians. The tops and bottoms of boxes represent the 75th and 25th percentiles, respectively. The upper and lower whiskers extend to data no more than 1.5× the interquartile range from the upper edge and lower edge of the box, respectively. **(C)** Unconstrained principle component analysis (for principal coordinates PC1 and PC2) with Bray–Curtis distance. **(D)** The microbiota composition of the three groups at the phylum level. **(E)** The microbiota composition of the three groups at the genera level.

A total of 2,851 species were annotated in the DENV2-infected, DENV2-uninfected, and control groups. All three groups shared 2,572 (90.21%) microbiotas. Approximately 71 species (2.75%) were shared between infected and uninfected groups. The microbiota exclusive to DENV2-infected accounted for 23 species (0.81%). There were 22 (0.77%) species of microbiota exclusive to the control group and 39 (3.17%) species exclusive to the uninfected group (Figure 1A).

Measurements of within-sample diversity (alpha-diversity) showed differences in microbiome between the three groups. Diversity indices are usually used to judge the stability of communities. The Shannon–Wiener index reflected the diversity of community species based on the number of species: an increase in the number of species in a community represented an increase in the complexity of the community. When the value of Simpson dominance index was larger, it mean, the uninfected mosquito group had the highest Shannon and Simpson index, indicating that exposure to DENV may increase the bacterial species of *A. albopictus*. The diversity of the microbiota of DENV2-infected mosquitoes was lower than that of DENV2-uninfected mosquitoes, suggesting that the susceptibility to DENV2 may be related to the diversity of bacterial species (Figure 1B). Besides, Ace index and Chao index were used to estimate the species richness with the number of OTUs in the community. DENV-uninfected mosquito had a higher Ace index and Chao index (2,448.7 and 2,433.5) than infected (2,434.5 and 2,428.5) and control mosquito (2,428.3 and 2,411.4), suggesting a greater number of OTUs in the community and the greater the community richness. Species richness and diversity were consistently higher in DENV uninfected mosquito.

The principal component analysis (PCA) revealed that the microbiota of DENV2-infected, DENV2-uninfected, and control separated in the first axis and formed three distinct clusters, with overlap in both groups exposed to the virus. Microbial abundance in the infected group was more similar to the uninfected group and differed significantly from the control group, indicating that the greatest source of variation in the microbiota was proximity to DENV2 exposure (Figure 1C).

In Figure 1D, the microbiota composition of the three groups was quite similar at the phylum level. The top 10 phyla accounted for more than 90% of all phyla. In particular, Proteobacteria and Ascomycota, which accounted for about 35%, respectively, had the highest relative abundance in the infected group (38.89, 34.56%) compared to the uninfected group (38.69, 34.45%) and the control group (38.83, 34.54%). Similarly, at the genus level, the top 30 genera accounted for more than 90% of the numbers, with *Aspergillus* being the most numerous at nearly 30%, followed by *Metarhizium* at more than 15% (Figure 1E).

### Random-Forest Model Detects Indicator Bacterial Families

**FIGURE 2:**
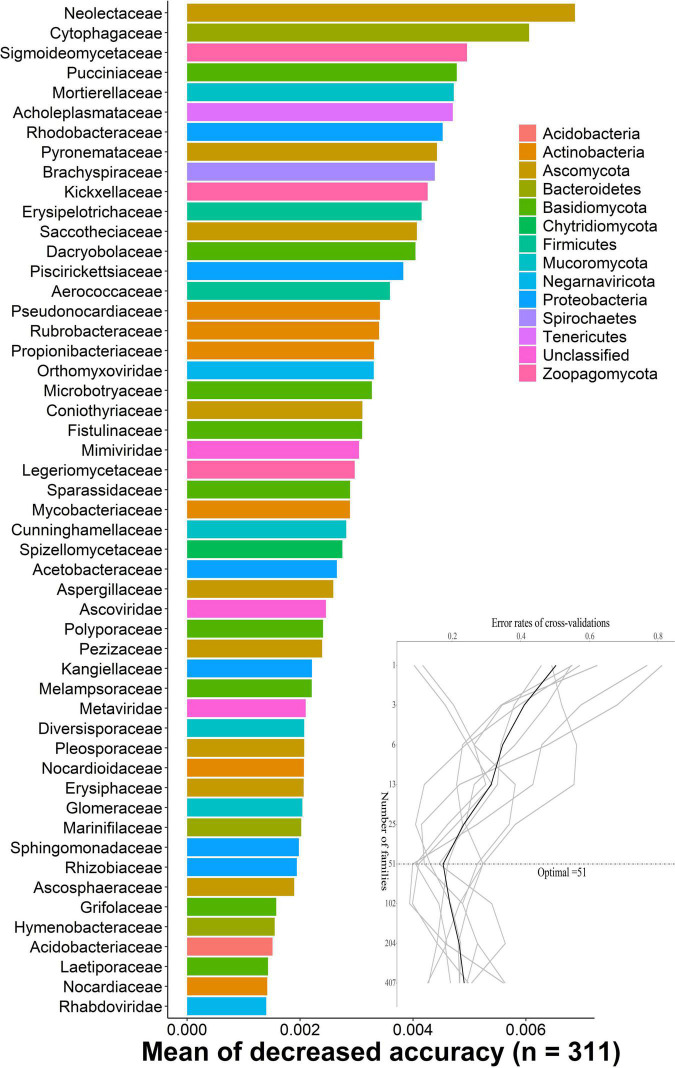
The random-forest model detects bacterial taxa that predict DENV infection. The possible biomarker taxa were ranked in descending order of importance to the accuracy of the model. The inset represents 10-fold cross-validation error as a function of the number of input families used to differentiate microbiota in order of variable importance.

The random-forest model detects bacterial families that predict DENV2-infected, uninfected, and not exposure to DENV mosquitoes. We carried out 10-fold cross-validation with five repeats to evaluate the importance of indicator bacterial families. The cross-validation error curve stabilized when the 51 most relevant families were used. Thus, we defined these 51 families as possible biomarker families. The top 51 bacterial families were identified by applying random-forest classification of the relative abundance of the microbiota. Biomarker families were ranked in descending order of importance to the accuracy of the model. Neolectaceae and Cytophagaceae were the most important microbial species for classification (Figure 2).

### Different on High Abundance Microbiota Between Three Groups of Mosquitoes

According to the row scaled heatmap, the microbial abundance of the control group (no exposure to DENV) is significantly different from the other two groups (exposure to DENV). Based on the abundance of the microbes, it can be seen that *Wolbachia* differed significantly from the other species, occupying a separate branch in the taxonomic tree. *Anaplasma* also occupied a separate branch of the taxonomic tree, was a microbe with a high percentage of abundance mentioned in the previous section (Supplementary Figure 2).

### Possible Microbiota Related With Dengue Fever Virus Susceptibility

We screened for possible microbiota related to DENV susceptibility by analyzing different microbiota between DENV2-infected and uninfected mosquitoes. Using metagenomeSeq method for susceptible and non-susceptible mosquitoes, 18 microbial species with high variation were screened, namely *Pseudomonas veronii*, *Acinetobacter* spp. CIP 102129, *Gammaproteobacteria*. The LefSe method revealed 15 differential organisms between susceptible and non-susceptible mosquitoes by analyzing the difference in abundance of microbes at the species level, of which 8 showed down-regulation, i.e., lower abundance in susceptible mosquitoes, and 7 showed up-regulation, i.e., higher abundance in susceptible mosquitoes. The functional annotation of the differential microbes is shown in Table 1. The results showed that in infected group microbes of the Chytridiomycetes phylum, Acholeplasmataceae family, Desulfovibrionaceae family, and *Desulfovibrio* sp. DS-1, *Enterococcus gallinarum*, and *Paenibacillus* sp. IHB B 3415 regulated significantly.

**TABLE 1:** Possible microbiota related with dengue fever virus (DENV) susceptibility to *Aedes albopictus.*

Name	Log2FC	*P*-value	Function	Regulation
*Culex* rhabdo-like virus	−4.38	0.0068	Unknown	Down
*Pinus nigra* virus 1	−2.918	0.0030	Transposon Tf2-12 polyprotein	Down
*Alphaproteobacteria bacterium* 65-37	−2.90	0.0154	Pyruvatedehydrogenase E1 component subunit beta mitochondrial	Down
*Candidatus Dadabacteria bacterium*	−2.60	0.0072	Adenosylhomocysteinase 1, non-specific lipid-transfer protein	Down
*Murmansk poxvirus*	−2.53	0.0042	Serine/threonine-protein kinase VRK1	Down
Methyloglobulus morosus	−2.52	0.0135	Glucosed dehydrogenase (FAD quinone)	Down
*Parcubacteria group bacterium* GW2011 GWA2 43 13	−2.50	0.0070	CAD protein	Down
*Nocardia* spp. YIM PH 21724	−2.46	0.0346	High affinity cationic amino acid transporter 1	Down
*Shigella sonnei*	2.46	0.0393	Uncharacterized protein ORF88	Up
*Betaproteobacteria HGW-Betaproteobacteria-5*	3.00	0.0446	Unknown	Up
*Thielavia terrestris*	3.22	0.0283	Unknown	Up
*Pseudorhodoferax soli*	3.25	0.0355	5-Methylthioadenosine/*S*-adenosylhomocysteine deaminase	Up
*Acinetobacter* spp.	3.27	0.0215	Unknown	Up
*Azospira* spp. I13	4.08	0.0049	Putative peptide zinc metalloprotease protein	Up
*Stanieria cyanosphaera*	4.26	0.0023	Apoptosis-inducing factor 3	Up

### Kyoto Encyclopedia of Genes and Genome Pathway Analysis

**FIGURE 3:**
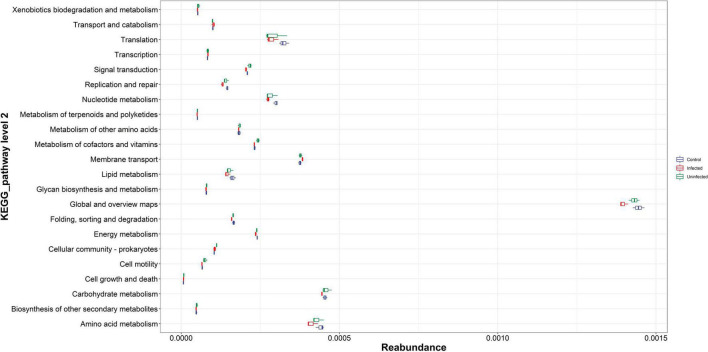
The Kyoto Encyclopedia of Genes and Genomes (KEGG) pathway abundance map. The vertical coordinates were the KEGG metabolic pathway or secondary metabolic pathway level classification; the horizontal coordinates were the relative content of the corresponding functional gene classification.

There were 22 pathways with relatively high abundance in KEGG, namely amino acid metabolism, biosynthesis of other secondary metabolites, and carbohydrate production. Among the above high abundance pathways, there were some differences in amino acid metabolism, carbohydrate metabolism, global and general overview maps, nucleotide metabolism, and translation. These differential pathways were relatively more abundant in the normal blood meal group than in the DENV2 exposed group, especially for global and overview maps with highest reabundance. Microbial metabolism in diverse environment like the citrate cycle was an important and conserved aerobic pathway for the final steps of the oxidation of carbohydrates and fatty acids. In this study, microbial metabolism like the citrate cycle was more enriched in uninfected mosquito; this implies that DENV infection may affect many biological processes, contributing to the disorder of microbiota to maintain metabolism (Figure 3).

## Discussion

Mosquito encountered diverse assemblages of bacteria (i.e., “microbiota”) and fungi from aquatic and terrestrial habitat. Individual mosquitoes in the same areas have radically different microbes, which are likely picked up from the environment. There were 158 species of fungi had been isolated from mosquito, nearly half of which could establish longer-term interactions with their hosts (Tawidian et al., 2019). The populations of symbiotic microbes changed depending on the species, period, and diet of the mosquito (Wang et al., 2011; Vogel et al., 2017). The main phyla of the *A. albopictus* microbiota were Proteobacteria, Ascomycota, Bacteroidetes, and Firmicutes, all of which had been similarly reported in other mosquito species (Pidiyar et al., 2004; Rani et al., 2009; Boissiere et al., 2012; Lin et al., 2021). Among the Proteobacteria phylum, two genera, *Wolbachia* and *Serratia*, were most associated with mosquitoes; *Wolbachia* stimulates the production of reactive oxygen species and activates the immune system in the body, resulting in the production of antimicrobial peptides to fight viruses (Pan et al., 2012; Mains et al., 2016). *Serratia* produced hemolysin, which facilitated the digestion of food after blood absorption (Gusmao et al., 2010). *Wolbachia* was closely related to the transmission of DENV and had been confirmed to inhibit the spread of dengue fever. *Wolbachia* also affects the vector competence of mosquitoes, and has been shown to be antagonistic to certain arboviruses in mosquitoes such as *A. albopictus*, *A. aegypti*, and *Aedes polynesiensis* and other mosquitoes (Moreira et al., 2009; Walker et al., 2011; Bian et al., 2013). Nazni et al. (2019) successfully introduced *Wolbachia* into mosquitoes to prevent dengue virus transmission, which will have promising applications in the field of mosquito vector and mosquito-borne disease prevention and control.

In our study, the fungal communities identified in mosquito carcasses were dominated by fungi of the phyla Ascomycota. This paralleled previous study which reported the composition and diversity of fungal communities associated with *A. albopictus* larvae and their natural habitats. It can be explained by the ubiquity of Ascomycota in freshwater ecosystems, including larval habitats (Tawidian et al., 2021). *Aspergillus clavatus* (Ascomycota: Trichocomaceae) was previously found to be an opportunistic pathogen of mosquitoes. Toxins secreted altered the larvae of the southern house mosquito, *Culex quinquefasciatus* larval tissues, leading to their necrosis and causing larval death (Bawin et al., 2016). There was a significant difference in the Chytridiomycetes phylum in DENV2-infected mosquito. According to previous literature, the Chytridiomycetes was found to be a host-specific parasite of mosquitoes, black flies, and ladybugs larvae (Whisler et al., 1975). By killing the mosquito and transferring to other organisms, it formed diploid methanogenic conidia through a series of proliferations, and the fertilized conidia transferred to larvae (Apperson et al., 1992). It was suspected that adult mosquitoes which infected Chytridiomycetes had a higher susceptibility to DENV2, allowing application to prevent the transmission of dengue virus by infecting adult mosquitoes with high levels of Chytridiomycetes.

It was hypothesized that the lack of a specific bacterium may lead to increase the susceptibility of mosquitoes to DENV. Approximately, 18 microbial species with high variation were screened for susceptible and non-susceptible mosquitoes, namely *P. veronii*, *Acinetobacter* spp. CIP 102129, *Gammaproteobacteria*, etc. Among them, *Gammaproteobacteria bacterium* has up to 110 corresponding functions in functional controls and most of them were associated with vital activities, which had been isolated by some scholars in mosquitoes (Wang et al., 2017; Wei et al., 2017; Wu et al., 2019), but there was still not much evidence to link it to dengue virus infection in mosquitoes. *Lactobacillus harbinensis* has been detected in the digestive system of *Anopheles culicifacies* (Sharma et al., 2014), and it was assumed that this microbe can help mosquitoes in digestion. Since the abundance of *L. harbinensis* increased after feeding on dengue virus, it is speculated that dengue virus may promote the amplification of *L. harbinensis*, which needed further experimental verification. The abundance of *Neurospora crassa* in the control group was significantly higher than that in the infected group, and it was speculated that dengue virus may inhibit its growth. It led to significant mortality in the presence of *Anopheles gambiae* defensin peptides (Vizioli et al., 2001). Several of the above microbes whose expression levels were up-regulated stimulation after DENV infection suggest their potential ability as an indicator for mosquito infection with dengue virus.

In this study, functions on possible microbiota related with DENV susceptibility to *A. albopictus* were screened. One of them was the transposon Tf2-12 polyprotein, a member of the retrotransposable (RTPs) family, whose main function was to encode a potential reverse transcriptase expressed in *A. aegypti* (Warren et al., 1997). Glucose dehydrogenase had been shown to play an important metabolic role in *Anopheles stephensi* (Mohanty et al., 2012). Other functions had not been reported in relation to mosquitoes.

## Conclusion

The dominant phyla of the *A. albopictus* microbiome were *Proteobacteria* and *Ascomycota*, and the major genera were *Aspergillus* and *Metarhizium*. *Gammaproteobacteria*, *L. harbinensis*, and *N. crassa* might serve as biomarkers of *A. albopictus* infection with Dengue 2 virus. Screening of microbes associated with DENV2 infection in *A. albopictus* revealed 15 different microbes, such as *Alphaproteobacteria bacterium* 65-37, *Methyloglobulus morosus*, and *Shigella sonnei*. The microbial functional analysis showed that they all correspond to proteins, enzymes, and other functional substances, basically involved in circulation, protein synthesis, substance breakdown, and other life-related enzymes that may be involved in the immunity and metabolism of the mosquito itself. It was hypothesized that the lack of specific bacteria may lead to increased susceptibility of mosquitoes to DENV. Artificial intervention in the microbiome composition or special bacterium in *A. albopictus* may alter the susceptibility to the DENV.

## Data Availability Statement

The datasets presented in this study can be found in online repositories. The names of the repository/repositories and accession number(s) can be found below: https://www.ncbi.nlm.nih.gov/bioproject/PRJNA777640.

## Ethics Statement

The animal study was reviewed and approved by the Institutional Ethics Committee of State Key Laboratory of Pathogens and Biosecurity/Institute of Microbiology and Epidemiology (protocol code IACUC-IME-2019-31).

## Author Contributions

ZL and C-XL: conceptualization. Y-QD: data curation. M-JL: formal analysis. TZ and B-QL: methodology. TZ: project administration and writing – review and editing. H-TG: software. DX: supervision. Y-EZ and ZL: validation. H-DZ: visualization. TZ, B-QL, and H-TG: writing – original draft. All authors contributed to the article and approved the submitted version.

## Conflict of Interest

The authors declare that the research was conducted in the absence of any commercial or financial relationships that could be construed as a potential conflict of interest.

## Publisher’s Note

All claims expressed in this article are solely those of the authors and do not necessarily represent those of their affiliated organizations, or those of the publisher, the editors and the reviewers. Any product that may be evaluated in this article, or claim that may be made by its manufacturer, is not guaranteed or endorsed by the publisher.

## Footnotes

1
https://www.ncbi.nlm.nih.gov/

## Supplementary Material

Supplementary Figure 1 The species accumulation curves assessing the quality of sequencing data.

Supplementary Figure 2 The heatmap of different high abundance microbiota between three groups of *Aedes albopictus*.
