# Supplementary material for: Metagenome Sequencing Reveals the Microbiome of Aedes albopictus and Its Possible Relationship With Dengue Virus Susceptibility
Source: Front Microbiol. 2022 May 11;13:891151. doi: 10.3389/fmicb.2022.891151 (PMC9130775; doi:10.3389/fmicb.2022.891151)
Supplement: Supplementary file 1 [file Table_1.docx]

**Supplementary Table 1.** Metagenome sequencing data quality

| **Sample pool ID** | **Number of Reads** | **GC(%)** | **Q20(%)** | **Q30(%)** |
| --- | --- | --- | --- | --- |
| Infected-1 | 44,213,089 | 38.93 | 97.71 | 93.45 |
| Infected-2 | 41,523,414 | 38.9 | 97.48 | 92.92 |
| Infected-3 | 47,179,819 | 38.91 | 96.57 | 90.96 |
| Uninfected-1 | 43,573,009 | 39.39 | 97.17 | 92.23 |
| Uninfected-2 | 42,062,718 | 39.75 | 96.33 | 90.53 |
| Uninfected-3 | 42,336,930 | 39.04 | 96.83 | 91.56 |
| Control-1 | 37,348,805 | 38.9 | 96.63 | 91.13 |
| Control-2 | 38,815,034 | 39.32 | 95.47 | 89.01 |
| Control-3 | 38,040,137 | 39.21 | 96.33 | 90.57 |
