# Supplementary figures and images for: Metagenome Sequencing Reveals the Microbiome of Aedes albopictus and Its Possible Relationship With Dengue Virus Susceptibility
Source: Front Microbiol. 2022 May 11;13:891151. doi: 10.3389/fmicb.2022.891151 (PMC9130775; doi:10.3389/fmicb.2022.891151)

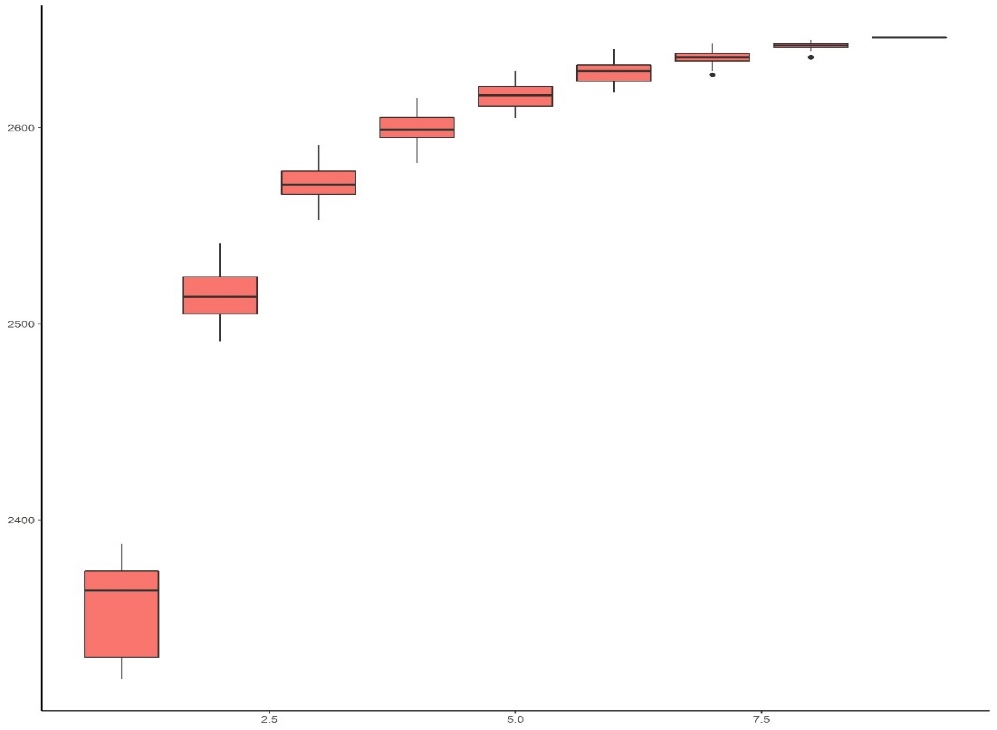

Supplement: Supplementary Figure 1 — The species accumulation curves assessing the quality of sequencing data. [file Image_1.JPEG]

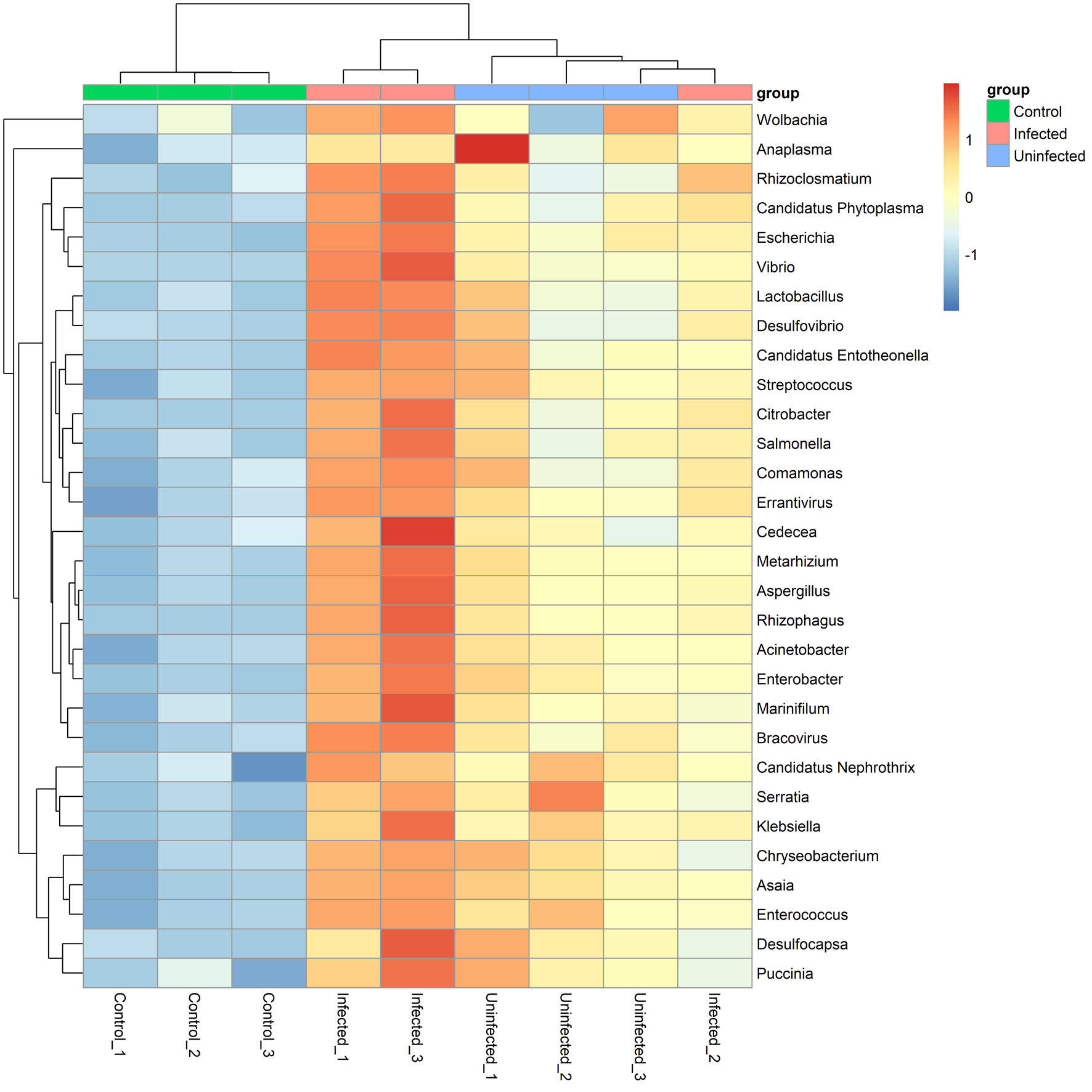

Supplement: Supplementary Figure 2 — The heatmap of different high abundance microbiota between three groups of Aedes albopictus. [file Image_2.JPEG]
